# Short- and long-term neurological outcomes of congenital cytomegalovirus infection

**DOI:** 10.55730/1300-0144.5819

**Published:** 2024-01-05

**Authors:** Fatih Mehmet Akif ÖZDEMİR, Yasemin TAŞÇI YILDIZ, Rüveyda GÜMÜŞER CİNNİ, Ayşegül ZENCİROĞLU, Deniz YÜKSEL

**Affiliations:** 1 Division of Pediatric Neurology, Department of Pediatrics, Faculty of Medicine, University of Health Sciences, Dr. Sami Ulus Maternity and Child Health and Diseases Training and Research Hospital, Ankara, Turkiye; 2 Division of Pediatric Radiology, Department of Radiology, Faculty of Medicine, University of Health Sciences, Dr. Sami Ulus Maternity and Child Health and Diseases Training and Research Hospital, Ankara, Turkiye; 3 Division of Pediatric Infectious Disease, Department of Pediatrics, Faculty of Medicine, University of Health Sciences, Dr. Sami Ulus Maternity and Child Health and Diseases Training and Research Hospital, Ankara, Turkiye; 4 Division of Neonatology, Department of Pediatrics, Faculty of Medicine, University of Health Sciences, Dr. Sami Ulus Maternity and Child Health and Diseases Training and Research Hospital, Ankara, Turkiye; 5 Division of Pediatric Neurology, Department of Pediatrics, Faculty of Medicine, University of Health Sciences, Dr. Sami Ulus Maternity and Child Health and Diseases Training and Research Hospital, Ankara, Turkiye

**Keywords:** Congenital cytomegalovirus infection, neurologic outcomes, magnetic resonance imaging

## Abstract

**Background/aim:**

Cytomegalovirus (CMV) is the most common congenital viral infection. Although most children with congenital CMV (approximately 85%–90%) are asymptomatic at birth, findings such as sensorineural hearing loss, microcephaly, and neurodevelopmental retardation can be observed during the follow-up. Among the brain magnetic resonance imaging (MRI) findings of CMV are white matter abnormalities, polymicrogyria, and periventricular calcification. Since a definitive diagnosis of congenital CMV cannot be made after the neonatal period, the identification of the associated phenotype is diagnostically important, but data are limited in patients who have been retrospectively diagnosed with congenital CMV infection. The aim of this study was to evaluate the short- and long-term neurological follow-up results of congenital CMV infections in a tertiary hospital.

**Materials and methods:**

The neurological results of fifteen patients under the age of 18 years, who had a definitive diagnosis of congenital CMV infection and were followed up in a tertiary care hospital between 2011 and 2020, were retrospectively evaluated.

**Results:**

Ten of the patients in our study group were male. The mean age at presentation for neurological evaluation was 2.02 ± 1.54 months, with a median follow-up time of 36.3 months (range: 9.3–129.4 months). Neurological disorders detected during the long-term follow-up included cerebral palsy (46.7%), cognitive impairment (46.7%), epilepsy (40%), and sensorineural hearing loss (26.7%). The most common abnormality observed on MRI scans was white matter involvement (53.3%).

**Conclusion:**

Early diagnosis and intervention are crucial in congenital CMV infection, as it commonly results in neurological involvement among the patients in our series. This preventable condition warrants further research regarding prenatal/neonatal screening.

## 1. Introduction

The overall incidence of congenital cytomegalovirus (CMV) infection varies between 0.5% and 3% in all live births [1]. Most infected children (85%–90%) are asymptomatic at birth [2]. However, CMV infection may manifest with symptoms or neurodevelopmental sequelae during the neonatal period. It has been shown that children born with an asymptomatic infection may subsequently develop sensorineural hearing loss (SNHL), cognitive deficits, and microcephaly [3]. Neurological signs play a crucial role in the early diagnosis, treatment, and rehabilitation, thereby reducing morbidity and mortality [4]. However, it is difficult to confirm CMV infection after the neonatal period. Clinical neurological, electrophysiological, and radiological characteristics, especially on brain magnetic resonance imaging (MRI), play a key role in cases of suspected congenital CMV infection. Typical MRI findings include white matter abnormalities, cerebellar hypoplasia, polymicrogyria, and periventricular calcification [5].

In this study, we aimed to determine the neurological profile of patients with a definitive diagnosis of congenital CMV infection (symptomatic or asymptomatic) who were followed up at a single tertiary care hospital and to evaluate their short- and long-term neurologic outcomes.

## 2. Methods

The patients included in this study were retrospectively screened from those registered with a referral diagnosis of P35.1, the International Classification of Diseases 10th Revision (ICD-10) diagnostic code for congenital CMV infection, at the University of Health Sciences, Dr. Sami Ulus Maternity and Child Health and Diseases Training and Research Hospital, Ankara, Türkiye, between 2011 and 2020.

The inclusion criteria were being under the age of 18 years, having a definitive diagnosis of congenital CMV, being followed up between January 2011 and December 2020, and having complete medical records. Exclusion criteria included any missing data in medical records and the inability to clearly differentiate between congenital and acquired CMV infection.

For patients with suspicious medical history and clinical findings, the diagnosis of congenital CMV infection was confirmed by performing virus cultures from urine, saliva, or blood and/or detecting viral DNA in the polymerase chain reaction (PCR) test conducted within the first two weeks of life [1].

For acquired CMV infection, viral culture or detection of CMV DNA in PCR tests of urine and saliva samples are the preferred diagnostic methods. However, negative results from urine or saliva samples collected within the first two weeks of life are necessary for diagnostic specificity. Without a negative result within the first two weeks of life, differentiation between congenital and acquired CMV is not possible [1]. We classified these patients as having probable congenital CMV infection.

Out of the 61 cases coded under P35.1 as a preliminary diagnosis, 16 were excluded from the study due to the negative result of their CMV PCR test or its unavailability. Among the remaining 45 cases, 26 were considered to have probable congenital CMV infection, while the remaining 19 had a definitive diagnosis of congenital CMV based on the specified criteria mentioned above. However, four of these 19 patients were excluded due to missing data or inadequate neurological outpatient follow-up (Figure).

Patient age at presentation, sex, the presence of parental consanguinity, clinical findings at presentation, antenatal, perinatal, and postnatal variables, maternal and neonatal history, motor and cognitive characteristics, hearing and vision findings, and the presence of developmental delays, epilepsy, microcephaly, and intrauterine growth restriction (IUGR) were recorded from the patients’ electronic records. Motor disability was assessed using the Gross Motor Function Classification System (GMFCS) based on the patients’ most recent motor evaluation.

All MRI images were analyzed based on the system described by van der Knaap [6]. Abnormalities in deep gray matter, cerebellum, and brainstem were reviewed by a radiologist in terms of any signal or structural abnormalities. Cerebellar hypoplasia was evaluated visually. Additionally, existing cranial computed tomography (CT) images was assessed, especially for calcification. Electrophysiological evaluations were conducted on electroencephalography (EEG) findings, visual-evoked potentials (VEPs), and brainstem auditory-evoked potentials (BAEPs).

### 2.1. Statistical methods

The data analysis was conducted using the SPSS statistical software. Categorical data were expressed as frequency and percentage values, and numerical data were expressed as mean and standard deviation.

## 3. Results

Fifteen patients with a definitive diagnosis of congenital CMV were included in the study. The main clinical characteristics of these patients are summarized in Table 1.

Cranial imaging was performed with MRI in 11 patients (73.3%) and CT in six (40%). The most common abnormality observed on MRI was white matter involvement that was present in eight patients (53.3%), followed by cortical gyral abnormalities in three patients (20%), cerebellar abnormalities in three (20%), brainstem abnormalities in two (13.3%), and deep grey matter involvement in one (6.7%). Calcification was observed in four (66.7%) of the six patients whose CT images were accessible. The main neuroimaging findings in our patients are presented in Table 2.

Electrophysiological evaluations consisted of EEG in eight patients, VEPs in eight, and BAEPs in eight. Focal epileptic findings were observed in five (62.5%) of the eight patients evaluated with EEG. In the evaluation of VEPs, unilateral prolongation was detected in 50% of the eight patients tested, while bilateral prolongation was observed in 12.5%. During the BAEP evaluation, there was unilateral prolongation in 12.5% and bilateral prolongation in 12.5% of the eight patients tested, while no significant waves were obtained from 12.5% of the patients.

Five patients who were asymptomatic at birth developed symptoms during the follow-up period, with a mean age of symptom onset calculated to be 5.73 ± 8.76 months. Five patients received ganciclovir therapy, and four received valganciclovir. The median follow-up time was 36.3 months (range: 9.3–129.4 months).

In terms of prognosis, we determined that during the long-term follow-up period, seven patients (46.7%) developed cerebral palsy, seven (46.7%) experienced cognitive deficits. Additionally, six patients (40%) developed epilepsy, and four (26.7%) suffered from SNHL. However, no cases of hemiplegia or mortality were observed. In the last examination, according to the GMFCS assessment, 10 cases (66.7%) were classified as level I, one (6.7%) as level II, and four (26.7%) as level V.

## 4. Discussion

In this retrospective crosssectional study involving 15 patients with a definitive diagnosis of congenital CMV infection, it was shown that the most common findings during the short-term neurological follow-up were developmental delay accompanied by pyramidal findings, and the most common adverse neurological outcomes during the long-term follow-up were cerebral palsy (46.7%), intellectual disability (46.7%), epilepsy (40%), and SNHL (26.7%). In the literature, congenital CMV infection has been reported to cause cerebral palsy in 90%, microcephaly in 53% to 70%, IUGR in 50%, and seizures in 37% of patients [7–9]. Consistent with this literature, we observed microcephaly in 53.3% of our patients, IUGR in 46.7%, and seizures in 40%.

Our patients, who had symptomatic congenital CMV infection, presented to the neurology department at a mean age of 2.02 ± 1.54 months. In comparison to a study in the literature that reported an average age of 20 months at presentation, the patients in our series had an early neurological follow-up [7].

SNHL was observed in 27% of the children in our series, which is consistent with the prevalence rates ranging from 9% to 50% reported in the literature [10]. The rate of chorioretinitis in our sample was 6.7%. In a prior study, Coats et al. [11] reported ocular abnormalities in 22% of the patients. Eye pathologies were observed at a lower rate in our study compared to that reported in the literature.

It has been reported that the results of MRI scoring systems are valuable for the prediction of prognosis **[5, 12]**. We reevaluated the MRI images of our patients according to the system defined by van der Knaap et al. **[6]**. We found that white matter involvement was the most common abnormality among the 11 patients who underwent MRI (72.7%).

In certain scoring systems in the literature, the presence of polymicrogyria serves as an indicator of the highest level of severity and is associated with the most severe consequences [5, 12]. The two patients with polymicrogyria in our study displayed cognitive developmental delays and were classified as GMFCS level V, which supports the literature evidence that polymicrogyria is an unfavorable prognostic factor.

Congenital CMV infection should be considered when polymicrogyria of unknown etiology is present. Of the other two cases at GMFCS level V in our series, one had both lissencephaly and schizencephaly, while the other had no cortical gray abnormality.

Among the eight patients detected to have white matter involvement in this study, the distribution of lesions was bilateral and multifocal in 37.5%, widespread and combined in 25%, and diffuse in 37.5%. The anterior temporal lobe was involved in four patients (36.4%). Cerebellar abnormalities were observed in three patients (27.3%) in our study, whereas previous studies have reported cerebellar hypoplasia in 40% to 70% of symptomatic CMV infants [13, 14]. This difference may be due to the degree and timing of brain damage. In comparison to the literature reports indicating white matter abnormalities in 100%, cortical malformation in 50%, and intracranial calcification in 48% of patients, our study found that white matter abnormalities and cortical malformation were less prevalent, while intracranial calcification was more common [15].

Van der Knaap et al. [6] reported that 75% of patients had abnormalities in the anterior temporal lobe and that this finding was specific to congenital CMV infection. Similarly, Kidokoro et al. [16] found a rate of 70%. In contrast, our study noted a relatively lower rate in (36.4%) compared to the literature. The differential diagnosis for this finding includes early-onset genetic leukoencephalopathies, such as Aicardi-Goutières syndrome and leukoencephalopathy with temporal lobe cysts [17].

Focal epileptic findings were detected in 62.5% of the eight patients evaluated by EEG in our study. Similar CMV isolation rates have been reported in healthy children and patients without EEG changes in the literature, whereas significantly higher isolation rates have been observed in groups with neurological and EEG abnormalities [18]. Only a few previous studies have evaluated the electrophysiological characteristics of patients with congenital CMV. However, focal EEG abnormalities are expected in patients with this condition, particularly those with cortical developmental abnormalities. In our study, among the eight patients evaluated for BAEPs, unilateral prolongation was noted in 12.5%, bilateral prolongation in 12.5%, while no wave was obtained in a further 12.5%. In the literature, it has been reported that urine viral load is associated with prolonged BAEP responses in neonates with CMV infection [19]. In our study, all patients with SNHL exhibited BAEP abnormalities, and SNHL was not observed in patients with normal BAEPs. These findings emphasize the need for electrophysiological evaluation in patients with congenital CMV.

Upon evaluating the patients’ prognosis, we found that during the long-term follow-up, cerebral palsy, cognitive delay, epilepsy, and SNHL were diagnosed in 46.7%, 46.7%, 40%, and 26.7% of the patients, respectively. However, hemiplegia or mortality was not observed in any case. In the last examination, based on the assessment of motor prognosis, 66.7% of the cases were classified as GMFCS level I, 6.7% as level II, and 26.7% as level V. In another study including 23 patients with symptomatic congenital CMV who developed cerebral palsy, 8.6% were classified as GMFCS level II, 8.6% as level III, 4.3% as level IV, and 78.3% as level V [20]. This difference can be attributed to the inclusion of both symptomatic and asymptomatic patients at birth in our sample, whereas the referenced study focused solely on those who developed cerebral palsy secondary to symptomatic congenital CMV infection. In another study involving patients with congenital CMV, who were followed up for a mean of 8.7 ± 5.3 years, the mortality rate was found to be 11.5% [21]. In our study, conducted over a mean follow-up period of three years, there were no occurrences of mortality. The same study also indicated that 65% of the patients were treated with ganciclovir, 38.4% of the patients developed cerebral palsy, 46.1% exhibited cognitive delay, 30.7% experienced epilepsy, 7.6% developed hemiplegia, and 38.4% developed SNHL. The higher rates of mortality and unfavorable neurological outcomes in that study can also be attributed to the authors’ inclusion of only patients with symptomatic congenital CMV infection [21].

The main limitations of this study include its retrospective design and the varying durations of patient follow-up. Due to the presence of clinical findings, such as developmental delay in one or more areas, hearing loss, and/or seizures in most patients in our study, the investigation of a patient group with different manifestations may yield a different frequency of MRI findings. However, the results of our study are significant because they display that CMV is still a common problem in the world and that patients may present to neurologists with varying clinical, electrophysiological, and radiological characteristics that may be confused with those of other neurological conditions.

## 5. Conclusion

Clinically, congenital CMV can manifest with cognitive and motor developmental delays, IUGR, neonatal cholestasis, microcephaly, seizures, anemia, ocular disorders, such as cataract and chorioretinitis, SNHL, and hepatosplenomegaly. Radiologically, congenital CMV infection should be considered in patients with bilateral periventricular and basal ganglion calcifications, white matter signal changes mimicking leukodystrophies, particularly cysts in the temporal and frontal lobes, as well as brain malformations associated with calcified cystic leukoencephalopathy and related calcifications. The understanding of these characteristic brain MRI and electrophysiological findings may support a clinical suspicion of congenital CMV infection, considering that a retrospective diagnosis beyond the neonatal period is usually not possible. Congenital CMV infection can lead to severe neurological morbidity, and most patients in our series also exhibited neurological involvement. Early diagnosis and intervention are crucial in this patient group, and this preventable disease warrants further research regarding prenatal and neonatal screening.

## Figures and Tables

**Figure:**
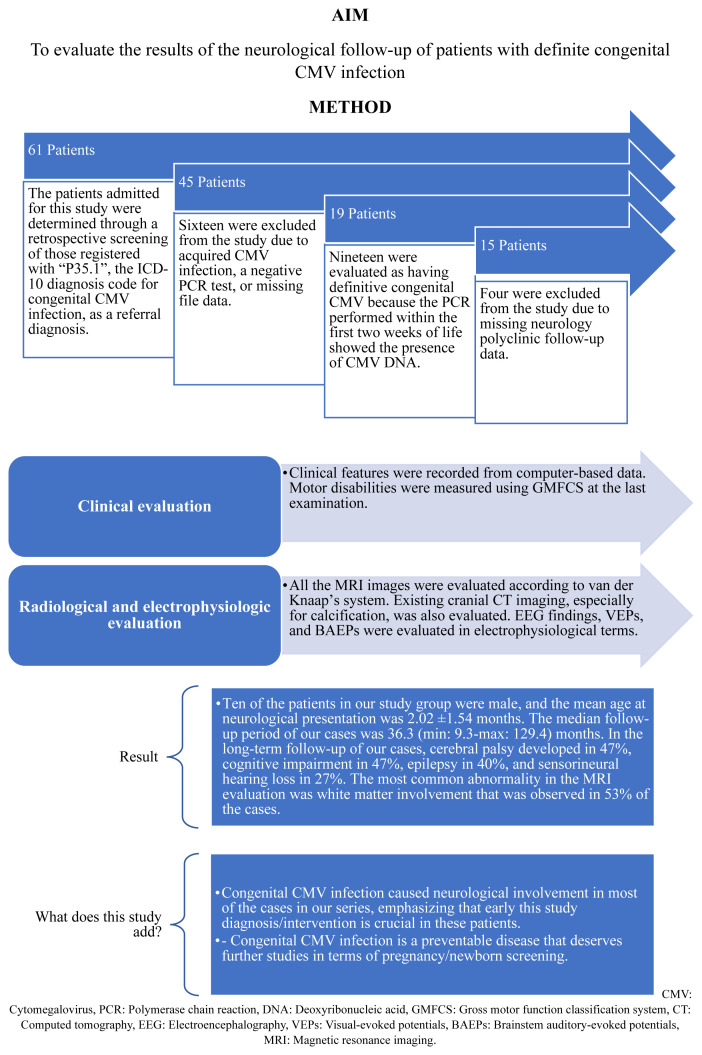
Study overview: short- and long-term neurological outcomes of congenital cytomegalovirus infection.

**Table 1:** Clinical characteristics of patients with congenital cytomegalovirus (CMV) infection.

	Definitive diagnosis of congenital CMV (n = 15)
Age at presentation to neurology, months (mean ± SD)	2.0 ± 1.5
Male	10 (67%)
Consanguinity	5 (33%)
CMV immunoglobulin M positivity	8 (53%)
Presence of prematurity	4 (27%)
Presence of IUGR	7 (47%)
Neonatal pneumonia	3 (20%)
Developmental delay	10 (67%)
Presence of seizure	6 (40%)
Dysmorphosis	4 (27%)
Microcephaly	8 (53%)
Serpigo: petechia/purpura	6 (40%)
Anemia	5 (33%)
Thrombocytopenia	7 (47%)
Jaundice	7 (47%)
Pyramidal findings	4 (27%)
Hemiplegia	0 (0%)
Splenomegaly	3 (20%)
Chorioretinitis	1 (7%)
SNHL	4 (27%)

CMV, cytomegalovirus; SD, standard deviation; IUGR, intrauterine growth retardation; SNHL, sensorineural hearing loss.

**Table 2:** MRI and neuroimaging characteristics of patients according to the van der Knaap scoring system.

	Confirmed congenital CMV (n)
**MRI features**	11
A) White matter abnormalities	8
1) Distribution of lesions: bilateral and multifocal/widespread and combined/diffuse	3/2/3
2) Symmetry of lesions: symmetric/asymmetric	6/2
3) Number of lesions: <10/10–20/>20	2/4/2
4) Consistency of lesion size: little variation in size/marked variation in size	8/0
5) Periventricular involvement	7
6) Deep white matter involvement	8
7) Arcuate fiber involvement	2
8) Weighted involvement: deep white matter/other	8/0
9) Frontal lobe involvement	4
10) Parietal lobe involvement	5
11) Occipital lobe involvement	3
12) Temporal lobe involvement	7
13) Location of largest lesions: parietal lobe/other	0/6
14) Myelination: normal/delayed	10/1
B) Cortical gyral abnormalities	3
1) Polymicrogyria	2
2) Symmetry: symmetric/asymmetric	2/1
3) Dominant location: lateral aspects/other	3/0
C) Deep gray matter abnormalities	1
D) Cerebellar abnormalities	3
E) Brainstem abnormalities	2
A) Anterior temporal lobe abnormalities	4
**CT features**	6
Calcification	4

MRI, magnetic resonance imaging; CMV, cytomegalovirus; CT, computed tomography.

## Data Availability

The authors have confirmed the availability of data and materials in this article.
